# Progress of Psf1 and prospects in the tumor: A review

**DOI:** 10.1097/MD.0000000000031811

**Published:** 2022-12-02

**Authors:** Xuekai Zhao, Botao Duan, Lei Zhou

**Affiliations:** a Department of Hepatobiliary Surgery, Binzhou Medical University Hospital, Binzhou, China.

**Keywords:** cancer, cell cycle, Gins complex, Psf1, tumor

## Abstract

Partner of Sld5-1(Psf1) is a member of Gins complex, which was discovered in 2003. It consists of the predominantly α-helical A-domain and the massively β-stranded B-domain. Some researches indicate that Psf1 plays a prominent part in DNA replication through cell cycle regulation, and plays a key role in early embryo development and tissue regeneration. The overexpression of Psf1 in active proliferating cells is closely correlated with the occurrence of tumors. On the side, tumor cells with high Psf1 expression showed high heterogeneity and poor clinical prognosis. In this review, we will review the research progress of Psf1 in cell cycle regulation, immature cell proliferation and oncology.

## 1. Introduction

Gins complex was first discovered by Takayama et al in gene screening of Saccharomyces cerevisiae in 2003. Gins is abbreviated from “go-ichi-ni-san,” which stands for Arabic number “5-1-2-3.” Gins is a heterotetrameric ring complex composed of 4 homologous subunits Psf1, Psf2, Psf3 and synthetically lethal with DNA polymerase B subunit (Dpb)11-1(Sld5).^[1]^ All proteins in the complex share 4 helical structures, and the core consists of Sld5 (residue 11-213), Psf1 (residue 1-151), Psf2 and Psf3.^[2]^ Gins complex is mainly involved in the initiation and extension of DNA replication in S phase of cell cycle, and plays an important role in the regulation of cell cycle.^[1]^ Psf1 and the other 3 subunits are highly conserved in the evolutionary process, and the relationship between Psf1 subunit and cell cycle regulation, immature cell proliferation and oncology is attracting more and more attention.

## 2. Structural characteristics of gins complex and psf1

In Gins complex, Sld5, Psf1, Psf3 and Psf2 combine clockwise to form an approximately symmetric structure, in which Sld5 is similar to Psf1 and Psf2 is similar to Psf3. From the overall structure, each subunit of Gins complex has the predominantly α-helical A-domain and the massively β-stranded B-domain. All proteins share 4 helices to form an arched and a jelly omella-like structure. Through sequence analysis, it was found that the sequence of 2 regions in Sld5/Psf1 subunit was “A-B,” but that in Psf2/Psf3 subunit was “B-A.”^[2]^

Psf1, also known as KIAA0186 and Gins1, human Psf1 gene is located on chromosome 20p11.21 and has 88% homology with mice. Psf1 gene contains 7 exons, encoding a protein consisting of 196 amino acids with a size of about 23KD. Psf1 has an arch structure formed by 4 helices H1, H2, H3 and H5, as well as B region rich in β crimp. Psf1 is connected with Sld5 and Psf3 in the overall structure. Moreover, the B region of Psf1 may be involved in the early process of DNA replication, and the position of Psf1 linker on the surface of nuclear complex plays many important roles in the replication activity. The conserved residues Phe173 and Leu205 in region B may be the sites of interaction with cell division cycle (Cdc)45, minichromosome maintenance (MCM) and chromatin, and the B domain of Psf1 may be more important functionally than the stability of the core complex.^[2,3]^

## 3. Gins complex and psf1 plays a prominent part in cell cycle

The cell cycle mainly consists of 2 stages: the first stage includes late M stage and early G1 stage, which is the preparation stage of DNA replication, and the level of cyclin-dependent kinases (Cdks) is low. The second stage mainly includes late G1 phase, S phase, G2 phase and early M phase, during which Cdk level is elevated and DNA replication and chromosome separation are completed.^[4]^ In eukaryotic cells, the initial process of DNA replication is a process participated by a variety of complexes and accurately regulated.^[5,6]^ At replication initiation, origin recognition complex (ORC), which consists of 6 subunits, first binds to the DNA replication initiation region ARSs. Then Cdc6 and Cdc10-dependent transcription (Cdt)1 interact with origin recognition complex (ORC) and combine with mini-chromosome maintenance (MCM) complex to form pre-replicative complex (pre-RC). In G1/S transition, pre-RC is transformed into pre-initition complexes under the action of Cdc45, and binding sites of Cdk and dbf4-dependent kinase (Ddk) appear. Subsequently, >8 factors including Cdk, dbf4-dependent kinase (Ddk), MCM10, Cdc45, Dpb11, Sld2, Sld3 and Gins participate in the activation process of MCM. Finally, the bidirectional replication fork is constructed to form the s-phase DNA replication capability.^[7]^

Gins, as 1 of the main components of replication, can bind DNA polymerase and enhance MCM activity during the transition from G1 to S phase of cell cycle. In S phase, Gins complexes bind to chromatin after pre-RCs formation, and this binding can be inhibited by P21 and Geminin. In addition, Gins complex interacts with Cdc45 and Dpb11/Cut5 in this binding process, but Gins complex has no direct effect on Cdc45 in this process.^[8–11]^ The research found that Gins complex forms CDC45-MCM2-7-GINS complex with MCM2-7 and Cdc45 in yeast, which regulates the origination of DNA duplication and the entire cell cycle. In mammalian cells, Gins complex participates in the DNA replication process in cells by binding to proteins related to DNA replication.^[12–15]^

Psf1, a key subunit of Gins’ role in the cell cycle, has been shown to be a highly conserved sequence. Researches have shown that Psf1 plays a prominent part in maintaining cell proliferation activity. After the expression of Psf1 was inhibited by RNAi, the DNA replication of cells was affected and the proliferation potential of cells was reduced, and the cell growth stagnated in S/G2/M phase, indicating that Psf1 could achieve the transition from G1/S to G2/M phase. Moreover, the expression of Psf1 gene was inhibited, and the spindle formation of the cells showed multipolarization. Chromosome segregation slowed down and abnormal segregation appeared, indicating that Psf1 also involved mitosis and chromatin assembly. The mechanism may be that the inactivation of Psf1 gene activates the key protein murine double minute (MDM)2 regulating the spindle, so the formation and function of the spindle are limited. At the same time, Survivin protein also appears in growth stagnant cells and inhibits the process of apoptosis.^[16]^ Further researches showed that the conserved residues Phe173 and Leu205 in Psf1 may be the sites of interaction with Cdc45, MCM and chromatin, and also play an important role in the activity of DAN polymerase ε.^[3,17]^

## 4. Psf1 and proliferation of immature cells

Psf1 stimulates cell proliferation and mitosis mainly by regulating the cell cycle, and plays a prominent part in the process of embryo development and tissue regeneration. Researches on mice found that Psf1 was generally expressed in developing mouse embryos. In adult mice, Psf1 was highly expressed in organs rich in proliferating cells, such as bone marrow, thymus, ovary, testis, etc., but could not be detected in mature differentiated liver, kidney, and brain (containing fewer mitotic cells). At the cellular level, Psf1 can be detected in many proliferative cells, but not in quiescent cells and mature differentiated cells. If Psf1 gene is knocked out, mouse endodermal cells will lose the ability to proliferate, leading to embryo stop growing and death before implantation.^[18]^ After mice were treated with 5-FU, hematopoietic stem cells in the bone marrow pool of Psf1+/- mice would show regenerative disorders and fail to prolifically rebuild in time, thus affecting the hematopoietic function of bone marrow.^[19]^ Researches have shown that Psf1 is a potential medium for the maintenance and activation of mouse hematopoietic stem cells, and its role is related to the negative regulation of histone acetylation mediated by E2F family factors.^[20]^ In researches of the proliferative vascular endothelial cells in tumor, Psf1 promoter activity can label proliferating cells in vivo, and the up-regulation of Psf1 promoter activity in endotheliocyte during angiogenesis was verified by detecting enhanced green fluorescent protein expression. Dormant endothelial cells in existing blood vessels expressed little or no Psf1.^[21]^ Researches on the role of Gins family protein in the development of mouse central nervous system demonstrated that the expression of Psf1 is connected with the proliferation of neural epithelial stem cells in the early stage of neural development.^[22]^ The above studies indicate that Psf1 is particularly important for the function of active proliferating cells (especially stem cells and progenitor cells).

## 5. Relevant investigation on psf1 and tumor

Tumor cells are similar to stem cells because of their proliferative potential. Recent reports have found that Psf1 plays a role not only in differentiated cells, but also in tumor cells.

Nagahama et al obtained the Psf1 gene promoter of mice through gene cloning technology, constructed a vector to monitor the expression of Psf1 by using the expression of enhanced green fluorescent protein, transfected it into mouse colon and lung cancer cells, and screened the cell lines stably expressing Psf1. It was found that the cell lines with high Psf1 expression were significantly stronger than the cell lines with low Psf1 expression in terms of tumorigenicity, invasion and metastasis. In vivo experiments, Psf1 was highly expressed in tumor cells around blood vessels, and GSEA analysis found that the expression of embryonic stem cell-like genes in tumor cells with high Psf1 expression significantly increased.^[16]^

In the research of breast cancer, Psf1 is less expressed in adjacent tissues and normal breast tissues than in cancer tissues. Moreover, survival analysis revealed that patients with low Psf1 expression had significantly longer survival than those with high Psf1 expression. Researches indicate that the level of Psf1 mRNA in normal epithelial cells was significantly lower than that in breast tumor cells. The detection of single nucleotide polymorphism did not find that the copy number of Psf1 gene increased, but the promoter activity of Psf1 gene increased. After the use of RNAi, the mRNA level of Psf1 in breast tumor cells decreased, and the proliferation potential of tumor cells decreased significantly due to the inhibition of DNA replication.^[23,24]^

In liver cancer tissues, Psf1 expression is strongly correlated with tumor grade, cirrhosis, vascular invasion and distant metastasis, and TNM staging. siRNA technology was used to downregulate Psf1 expression, and it was found that the invasion ability of cells was weakened after inhibiting Psf1 expression, which further indicates that Psf1 can promote the invasion ability of liver cancer.^[25]^

Zhang et al verified the correlation between Psf1and lung cancer through in vitro experiments. Compared with adjacent tissues, Psf1 was overexpressed in lung cancer samples. Downregulating the expression of Psf1 in lung cancer cell lines with different p53 gene backgrounds can successfully inhibit the proliferation of lung cancer cells and cause cell cycle arrest in a p53 independent manner. This experiment provides evidence that Psf1 may become a new target for lung cancer treatment^.[26]^

Researches have shown that Psf1 is also strongly expressed in high-grade prostate cancer. The expression of Psf1 is significantly correlated with tumor grade and clinical stage. In addition, its expression was also significantly correlated with the overall survival rate of patients with prostate cancer. The data showed that the cancer-specific survival rate of Psf1 positive group was significantly worse. It can be concluded that Psf1 may be a useful biomarker in prostate tumors, which can predict the prognosis of patients and optimize treatment decisions.^[27]^

Psf1 is also strongly expressed in non-small cell lung cancer, and the proliferation of lung cancer cells can be inhibited by downregulating the expression of Psf1. Psf1 is a prognostic marker for NSCLC patients receiving surgical treatment after preoperative chemotherapy or radiotherapy and chemotherapy. The research of Kanzaki et al did not find Psf1 expression in normal lung tissues, suggesting that drugs that inhibit Psf1 expression or function may be effective anticancer drugs.^[28]^

In the research of Psf1 on synovial sarcoma, L. Tang et al indicated that Psf1 expression was obviously increased in synovial sarcoma and was strongly correlated with prognosis. Downregulation of Psf1 expression by technology can inhibit the proliferation of synovial sarcoma cells and lead to apoptosis of cancer cells. Patients with high Psf1 expression in synovial sarcoma have a poor prognosis, and Psf1, as a downstream target gene of Anlotinib, may be a potential therapeutic target for synovial sarcoma.^[29]^ In addition, researches have indicated that Psf1 expression is also significantly upregulated in melanoma, colon cancer and intrahepatic cholangiocarcinoma.^[30,31]^

## 6. The prospect of psf1 in tumor research

Gins complexes play an important role in cell cycle, DNA replication and cell cycle disorder is a central link in genome instability, particularly in S phase of DNA replication, DNA damage will occur if regulation disorder, abnormal DNA replication, etc., the results make unstable further increased the possibility of canceration of the cells, resulting in the occurrence and development of tumor. The close connection between Psf1, as a core subunit, and cell cycle is worthy of further discussion, and the potential threat of tumorigenesis can be further explored by studying its regulation mechanism with eukaryotic cell cycle. It is noteworthy that Gins complex and Psf1 regulate the cell cycle in the nucleus, but in the study of mice, Psf1 expression in the cytoplasm of mouse blastocysts and transfected NIH3T3 cells was significantly higher than that in the nucleus.^[18,31]^

In the research of glioblastoma, Tokuhiro Kimura pointed out that the quiescence and proliferation of cancer cells are reversibly determined by the microenvironment inside and outside the niche. The proliferation activity was parallel to the expression level of Gins component, and the dynamic control of Gins component could make the tissue microenvironment decide the proliferation state of cancer cells. Inhibit its induction may be a prospective therapeutic strategy for controlling glioblastoma development and relapse.^[32]^ This suggests that Gins complex and Psf1 subunit may play a role in other cellular biological functions in eukaryotic cells besides cell cycle regulation.

Recently, the research on Psf1 as a therapeutic target is also worthy of attention. Mari Yoshida and others washed Psf1 peptides from affinity purified human leukocyte antigen (HLA) by mass spectrometry. These peptides induce Psf1-specific cytotoxic T lymphocyte responses, such as interferon-γ production and cytotoxicity. This research suggests that Psf1 polypeptide inoculation may be a new strategy for cancer treatment.^[33]^ In the researches on the drug resistance of leukemia cells, it is pointed out that Psf1 high expression is the most chemically resistant. Knockout of Psf1 gene in leukemia cells will lead to changes in the position of cancer cells in the distance to blood vessels. These findings may reflect the mechanism of leukemia cells escaping from chemotherapy and suggest that Psf1 may be a therapeutic target to enhance the effect of chemotherapy.^[34]^

On the other hand, tumor cells have the characteristics of infinite proliferation and low differentiation, and organs, tissues and cells with high Psf1 expression all show high proliferative potential. Meanwhile, tumor cells with high Psf1 expression are significantly stronger than cells with low Psf1 expression in proliferation, invasion and metastasis. Psf1 provides us with new research directions in terms of stem cell-like characteristics and its relationship with tumor heterogeneity, as well as whether Psf1 can be used as a therapeutic target for various tumors. In addition, whether Wnt, Notch and other stem cell-related pathways are targeted to Psf1 expression is also worthy of further discussion.

In summary, the important regulatory role of Psf1 in the cell cycle and its stem cell-like characteristics have opened up a new field for our research in tumor. Therefore, with the deepening of relevant research, Psf1 and Gins complex are expected to become an important index for the diagnosis, treatment and prognosis of malignant tumors in oncology, and may become a new target for more tumor treatment.

## Author contributions

**Writing—original draft**: Xuekai Zhao, Botao Duan.

**Writing—review & editing**: Lei Zhou.
